# Dietary glycerol monolaurate mitigates heat stress-induced disruption of intestinal homeostasis and hepatic lipid metabolism in laying hens

**DOI:** 10.1007/s44154-025-00243-8

**Published:** 2025-08-12

**Authors:** Jiang Gao, Hongrui Ren, Xuanfu Wu, Cunzhi Zou, Bin He, Wenqiang Ma

**Affiliations:** 1 Key Laboratory of Animal Physiology and Biochemistry, Ministry of Agriculture and Rural Affairs, College of Veterinary Medicine, Nanjing Agricultural University, NO.1 Weigang Road, Nanjing, Jiangsu 210095 China; 2 MOE Joint International Research Laboratory of Animal Health & Food Safety, Nanjing Agricultural University, Nanjing, Jiangsu 210095 China

**Keywords:** Heat stress, Laying hens, Lipid metabolism, Intestinal barrier, Metabolomics, Transcriptomics

## Abstract

Heat stress (HS) disrupts intestinal homeostasis and hepatic lipid metabolism in poultry, yet effective interventions remain limited. We investigate the protective effects of dietary glycerol monolaurate (GML) supplementation in laying hens under HS conditions. In a 10-week trial, 504 Hy-Line Brown hens were assigned to four groups (control and GML at 65, 195, and 325 mg/kg) with six replicates per group. Hens receiving 325 mg/kg GML exhibited significantly higher egg production and egg weight (*P* < 0.05), alongside improved egg quality metrics, including increased shell strength and Haugh units by week 8 (*P* < 0.05). Histological analysis revealed that GML (325 mg/kg) improved duodenal and ileal villus height and duodenal villus-to-crypt ratios while reducing duodenal crypt depth (*P* < 0.05), thereby restoring gut barrier integrity. These findings were supported by reduced plasma D-lactate (D-LA) levels and upregulated expression of tight-junction proteins ZO-1 and Occludin in the ileum and jejunum (*P* < 0.05). In the liver, GML supplementation alleviated HS-induced steatosis, reducing lipid droplet accumulation (*P* < 0.05), plasma low-density lipoprotein cholesterol (LDL-C), aspartate aminotransferase (AST), and alanine aminotransferase (ALT) levels, and hepatic triglyceride content, while elevating high density lipoprotein cholesterol (HDL-C). Integrated plasma metabolomics and hepatic transcriptomics identified 36 differential metabolites (enriched in sphingolipid metabolism) and 1,176 differentially expressed genes (enriched in PPAR signaling and Fatty acid degradation), with *ACSL1* as a central regulatory gene. Key genes (*ACSL1*, *CPT1 A*) and metabolites correlated positively with production performance and gut-liver health, while *SCD* and Probucol showed negative associations. These findings indicate that GML supplementation enhances intestinal barrier function, promotes hepatic fatty acid β-oxidation, and reinforces sphingolipid metabolism, thereby mitigating HS-induced oxidative stress and lipid dysregulation. Our results identify 325 mg/kg GML as the optimal dosage, proposing a practical strategy to enhance poultry resilience during heat stress.

**Supplementary Information:**

The online version contains supplementary material available at 10.1007/s44154-025-00243-8.

## Introduction

Heat stress represents a critical challenge in poultry production, as global temperatures rise, the poultry industry faces escalating risks to productivity and animal welfare, underscoring the urgency of identifying management solutions to mitigate heat-induced declines and enhance resilience in commercial flocks (Mack et al. 2013; St-Pierre et al. 2003). HS induces multifaceted physiological disruptions in poultry, manifesting as electrolyte imbalance, increased oxidative stress, and endocrine dysregulation, these systemic perturbations are accompanied by compromised intestinal barrier integrity and disrupted hepatic lipid homeostasis. Consequently, these pathophysiological alterations significantly impair the productive performance of laying hens, primarily through suppressed feed intake, ultimately resulting in diminished egg production and compromised egg quality (Kikusato et al. 2023; Mashaly et al. 2004; Lara et al. 2003). Compared to mammals, poultry have less efficient thermoregulatory mechanisms, such as the absence of sweat glands (Chowdhury et al. 2012), relying on panting for heat dissipation. Prolonged panting can disturb CO_2_ homeostasis by lowering plasma bicarbonate levels. This, combined with alterations in carbonic anhydrase activity, can result in respiratory alkalosis (El Hadi and Sykes 1982). Exposure to heat stress reduces intracellular Ca^2+^ levels and opens electrolyte channels, which along with decreased cell permeability, predisposes cells to apoptosis. Additionally, acute heat stress increases malondialdehyde (MDA) levels in both plasma and mitochondria of chickens (Mujahid et al. 2007). Elevated MDA levels indicate increased reactive oxygen species (ROS), leading to severe oxidative damage and imposing a significant burden on the liver of laying hens, making it more susceptible to damage. Ambient temperatures above 30 °C can decrease egg yield by 15% ~ 20% and negatively affect egg quality, including egg weight, yolk color, eggshell thickness, nutritional value, and shelf life, while also increasing mortality rates in commercial flocks (Lara and Rostagno 2013; Saeed et al. 2019). During severe HS, the increase of ROS production in mitochondria exceeds the antioxidant reserve, leading to oxidative damage of protein, lipid and DNA, which in turn leads to the reduction of ATP synthesis and dysregulation of calcium metabolism (Akbarian et al. 2016). HS significantly impacts nutrient absorption, contributing to the decline in production performance. HS alters digestive enzyme activity and transporter expression, disrupting nutrient absorption, utilization, and metabolism in the intestine. These disruptions can lead to intestinal pathologies, compromise epithelial barrier integrity, and ultimately result in impaired growth performance indices. (Varasteh et al. 2015; Feng et al. 2012; Chen et al. 2014), the increase of ROS level during HS leads to the decrease of intestinal volume, which improves the permeability of the intestinal tract, leading to bacterial translocation from the intestinal tract (Lara and Rostagno 2013).

Conventional methods using electrolytes (e.g., bicarbonate) and direct antioxidants (e.g., vitamins E/C) provide temporary relief but fail to address the root causes of HS pathology, such as ongoing gut barrier dysfunction and hepatic lipidome dysregulation (Renaudeau et al. 2012). The disrupted PPARα/γ signaling and altered gut-liver axis communication under heat stress require targeted modulation of lipid metabolism. Oxidative stress induces membrane lipid peroxidation, functional protein modification, and genomic damage, triggering a cascade of events that progressively impairs cellular physiology (Pizzino et al. 2017). Antioxidants can inhibit ROS accumulation, but they cannot restore the damaged intestinal barrier or regulate the disrupted liver lipid metabolism (Liu et al. 2018). Heat stress causes gut microbiota imbalance and metabolic disruption, particularly in branched-chain amino acids and short-chain fatty acids. This promotes hepatic lipid accumulation and leads to non-alcoholic fatty liver disease (NAFLD). Pure antioxidants alone can’t intervene in this process (Wen et al. 2021). Furthermore, the systemic effects of HS on hepatic lipid metabolism are complex and multifaceted. Heat stress promotes hepatic steatosis through the dual mechanisms of enhanced lipid synthesis and impaired lipid oxidation pathways, this metabolic dysregulation is frequently associated with the upregulation of pro-inflammatory mediators, particularly *TNF-α* and *IL-1β*, creating a vicious cycle that amplifies hepatocellular injury (Tang et al. 2021). This therapeutic impasse underscores the need for next-generation interventions targeting both intestinal barrier restoration and hepatic lipid metabolism reprogramming. These dual approaches address critical components of the gut-liver axis dysfunction induced by heat stress.

Medium-chain fatty acids (MCFAs) function as energy substrates and modulate glucose and lipid metabolism, endocrine signaling, and intestinal barrier function by regulating the gut microbiota (Nimbkar et al. 2022; Huang et al. 2021). While many functions of MCFAs have been confirmed, their specific effects on intestinal-liver axis remodeling in poultry, particularly regarding the reprogramming mechanism of sphingolipid metabolism, remain unclear. Medium chain fatty acids have the potential to be used as nutritional additives. Glyceryl monolaurate (GML), a naturally occurring medium-chain fatty acid abundant in human breast milk and coconut oil, is recognized by the U.S. Food and Drug Administration as a generally recognized as safe (GRAS) compound, this bioactive agent exhibits well-documented antimicrobial, antiviral, and anti-inflammatory properties (Mo et al. 2019). GML maintain mucosal barrier and promote the expression of intestinal barrier related genes *ZO1* and *Occludin* (Kong et al. 2021). Treatment with 1600 mg/kg GML resulted in a 50% reduction in LDL-C levels, while HDL-C concentrations showed a 36% increase relative to control values, though this difference did not reach statistical significance (Mo et al. 2019). GML can significantly improve lipid metabolism, reduce lipid accumulation and improve liver condition (Wein et al. 2009; Nagao and Yanagita 2010). The protective effect of GML on liver health is mainly through improving the antioxidant capacity and lipid metabolism of liver (Lin et al. 2022), previous studies have shown that GML can activate the AMP-activated protein kinase (AMPK) pathway. This activation reduces lipid synthesis in the liver and enhances lipid decomposition. GML helps alleviate lipid accumulation and liver damage caused by a high - fat diet. (Wang et al. 2022). The above research shows that GML can improve the health of livestock and poultry by affecting the gut - liver axis of the body, but the specific mechanism of GML as a nutritional additive for laying hens to resist heat stress needs to be further explored.

This study aims to investigate the potential of GML supplementation in alleviating HS-induced declines in egg production and quality in laying hens. Specifically, we focus on its role in restoring intestinal barrier integrity and rectifying hepatic lipid metabolic dysregulation. By integrating performance metrics, egg quality analysis, and multi-omics approaches, this study seeks to establish GML as a multifunctional feed additive for improving poultry resilience and productivity under HS conditions.

## Results

### GML enhances production performance and egg quality under heat stress

**Fig. 1:**
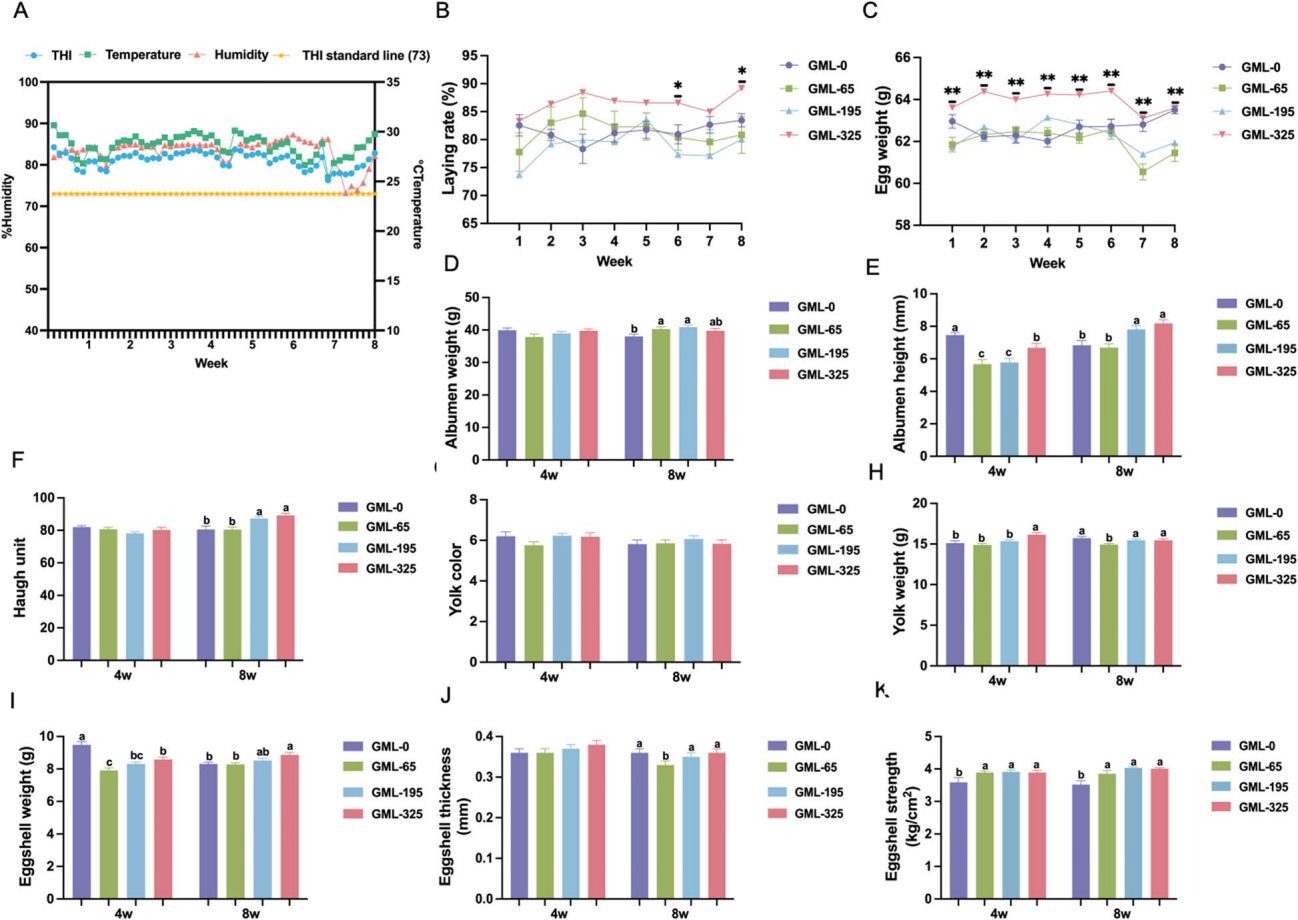
Effects of different concentrations of GML on production performance and egg quality of laying hens under heat stress. **A** Temperature and humidity index curve. **B** Effects of different concentrations of GML on laying rate and **C** egg weight, and **D** albumen weight, and **E** albumen height, and **F** haugh unit, and **G** yolk color, and **H** yolk weight, and **I** eggshell weight, and **J** eggshell thickness, and **K** eggshell strength. Data are presented as mean ± SEM, **B** and **C**, *n*=6; **D**-**K**, *n*=25. The *P* value is calculated by one-way ANOVA and Duncan’s multiple comparisons test. Different lowercase letters in the shoulder label indicate a significant difference (*P*<0.05)

Chronic heat stress (HS) was confirmed across the trial, with temperature-humidity index (THI) values consistently exceeding 73, far higher than the comfortable ambient temperature and humidity of laying hens (Fig. 1A). Supplementation with glycerol monolaurate (GML) at 325 mg/kg markedly improved production metrics relative to controls, increasing egg production (Fig. 1B, *P*< 0.05) and egg weight (Fig. 1C, *P*< 0.01). Egg quality exhibited time-dependent responses: albumen height decreased in GML-treated groups at week 4 (*P* < 0.05) but rose in GML-195 and GML-325 groups by week 8 (Fig. 1D, *P* < 0.05), mirrored by increased albumen weight in GML-65 and GML-195 at termination (Fig. 1E, *P* < 0.05). Haugh units followed a similar trend in GML-195 and GML-325 groups by week 8 (Fig. 1F, *P* < 0.05). Yolk weight increased acutely in GML-325 at week 4 (*P* < 0.05) but declined in GML-65 by week 8 (Fig. 1H, *P* < 0.05), with yolk color unchanged (Fig. 1g). Shell parameters showed dynamic shifts: shell weight dropped initially in GML groups at week 4 (*P* < 0.05) but increased in GML-325 by week 8 (Fig. 1I, *P* < 0.05), while shell thickness decreased in GML-65 at termination (Fig. 1J, *P* < 0.05). Eggshell strength was consistently elevated across all GML groups throughout (Fig. 1K, *P* < 0.05).

### GML restores intestinal morphology in heat-stressed hens

**Fig. 2:**
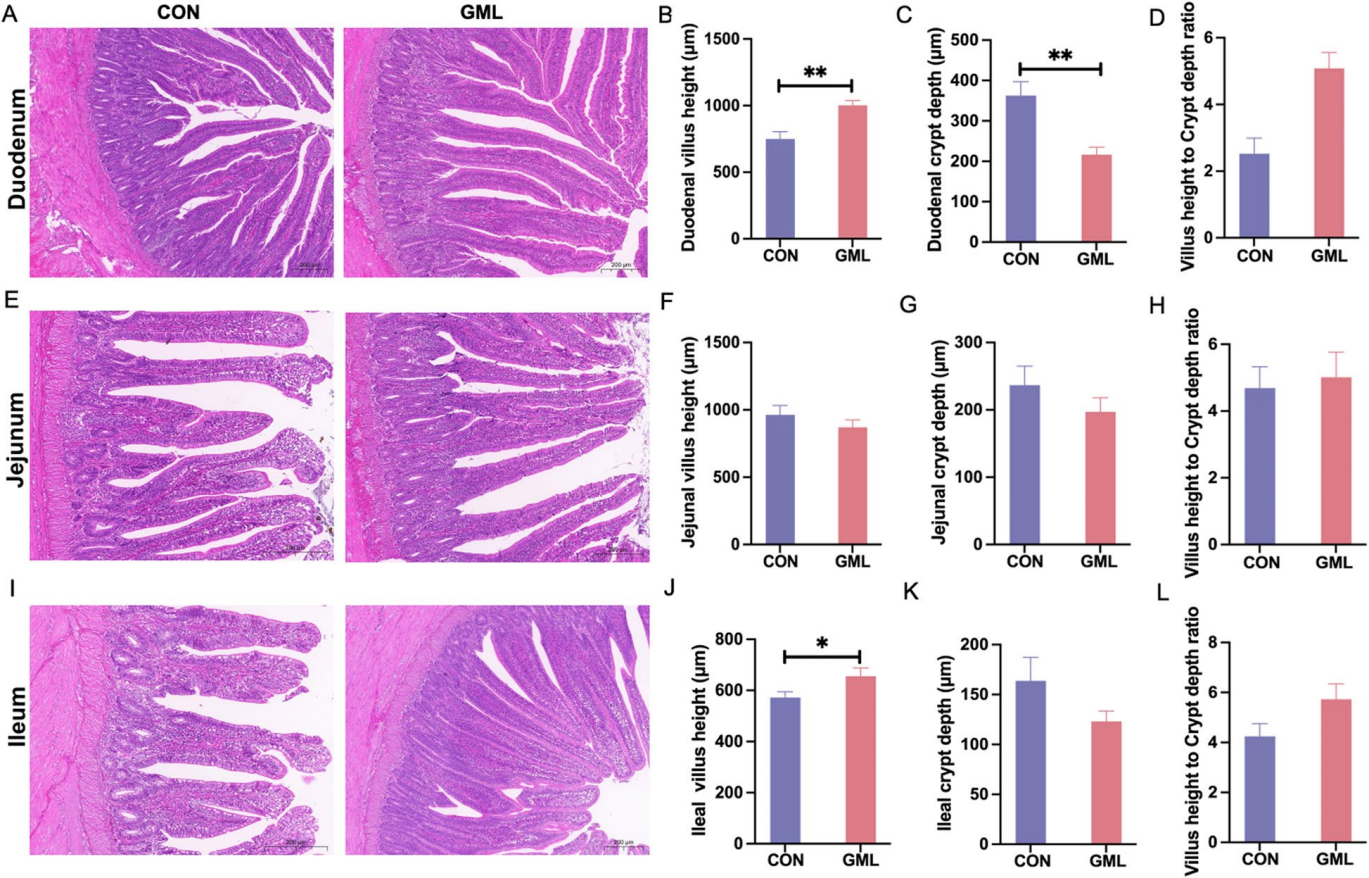
Effects of GML on small intestinal morphology in laying hens under heat stress. **A** H&E-stained sections of the duodenum. **B - D** Duodenal morphological indicators. **E** H&E-stained sections of the jejunum. **F - H** Jejunal morphological indicators. **I** H&E-stained sections of the ileum. **J - L** Ileal morphological indicators. Scale bar = 200 μm. The *P* value is calculated by student's *t* test and two-tailed. Data are presented as means ± SEM, *n*=6. * *P*<0.05, **P<0.01

Histological analysis revealed HS-induced damage in control group intestines, with duodenal villi showing shedding and inflammation (Fig. 2A). In contrast, GML-treated duodena appeared intact, with significantly increased villus height and villus-to-crypt ratios, alongside reduced crypt depth (Fig. 2B - D, *P* < 0.05). Jejunal crypts in controls exhibited epithelial disruption, unlike the GML group (Fig. 2E). Ileal villus height also rose in the GML group (Fig. 2J, *P*< 0.05), despite persistent inflammation and villi breakage in controls (Fig. 2I).

### GML bolsters intestinal barrier integrity

**Fig. 3:**
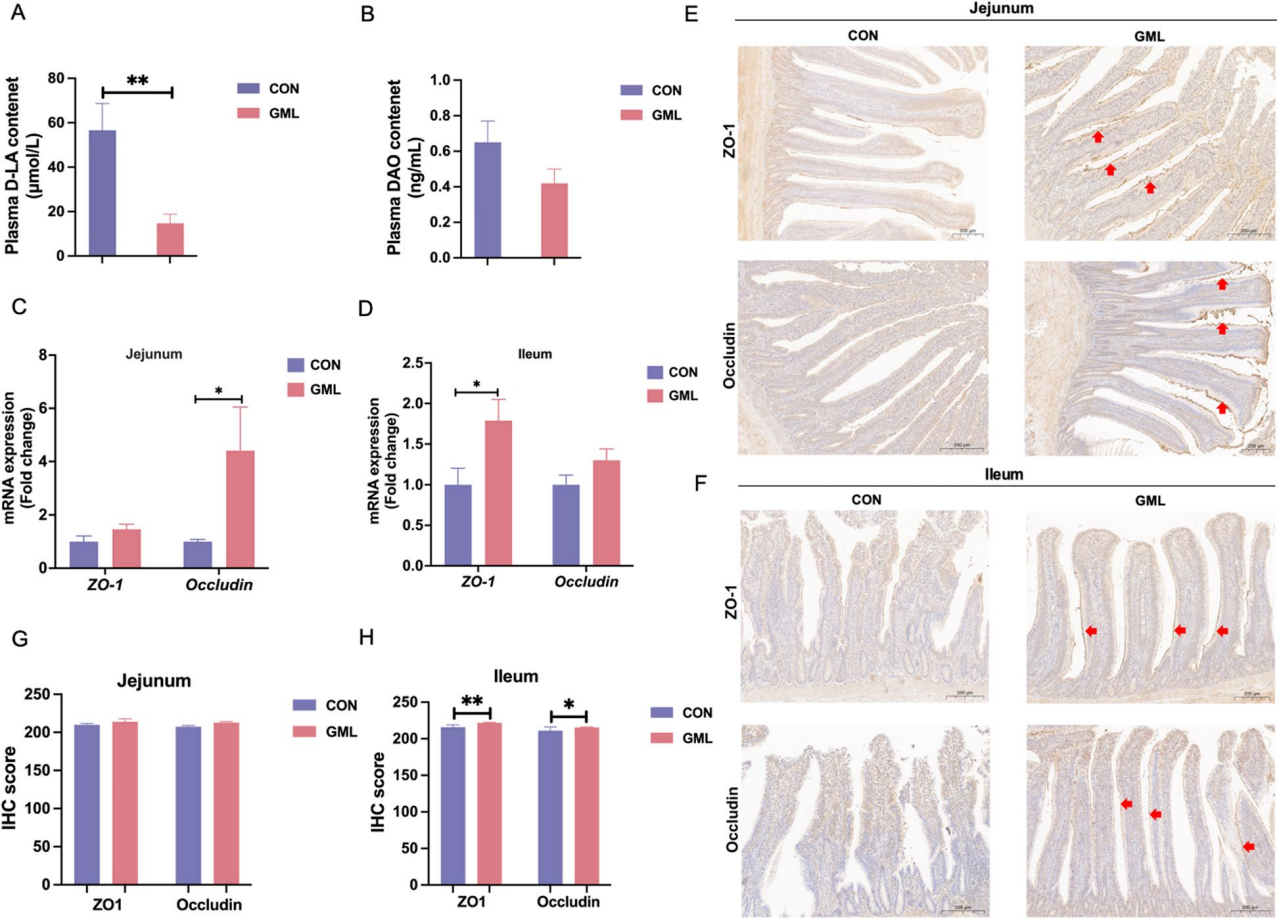
Effects of GML on intestinal barrier in laying hens under heat stress. **A** and **B** Plasma D-LA and DAO content. **C** and **D** Detection of *ZO-1* and *Occludin* mRNA expression in jejunum and ileum by qPCR. **E** and** F** Immunohistochemical staining for ZO-1 and Occludin in jejunum and ileum. **G** and** H** IHC score for ZO-1 and Occludin in jejunum and ileum. Scale bar = 200 μm. The *P* value is calculated by student's *t* test and two-tailed. Data are presented as means ± SEM, *n*=6. * *P*<0.05, **P<0.01

GML supplementation lowered serum D-lactate levels, a marker of gut permeability (Fig. 3A, *P* < 0.01). Gene expression of tight-junction proteins was upregulated, with ZO-1 elevated in the ileum (Fig. 3C, *P* < 0.05) and Occludin in the jejunum (Fig. 3D, *P* < 0.05). Immunohistochemistry confirmed increased ZO-1 and Occludin protein levels in GML-treated ileum (Fig. 3E - G, *P* < 0.05), supporting enhanced barrier function.

### GML mitigates hepatic steatosis and lipid dysregulation

**Fig. 4:**
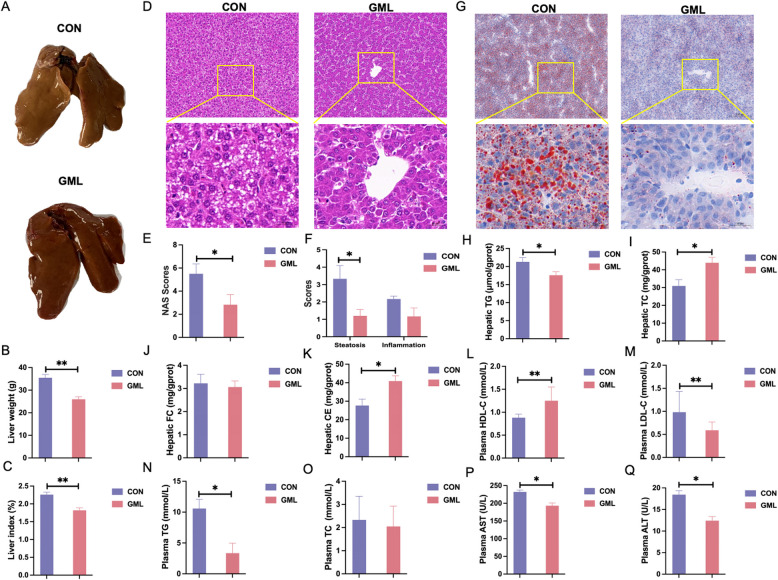
Effect of GML on liver lipid metabolism in laying hens under heat stress. **A** Gross morphology of the liver. **B** and **C** Liver weight and index. **D** H&E-stained sections of the liver. **E** NAS Score. **F** Histological scores for steatosis and inflammation. **G** Oil Red O-stained sections of the liver. **H - K** Hepatic lipid levels. **L - O** Plasma lipid levels. **P** and **Q** Liver injury indicators. The *P* value is calculated by student's *t* test and two-tailed. Data are presented as mean ± SEM, *n*=6. * *P*<0.05, **P<0.01

Hepatic tissue analysis revealed distinct morphological differences: the control group exhibited characteristic signs of lipid accumulation with yellowish discoloration and pronounced steatotic changes. In contrast, livers from GML group maintained their typical architecture without observable lipid infiltration (Fig. 4A). GML decreased liver weight, and liver-to-body ratio (Fig. 4B and C, *P* < 0.05). Hepatic HE and Oil Red O staining showed fewer lipid droplets in the GML group (Fig. 4D and G), with reduced NAS scores and steatosis severity (Fig. 4E and F, *P*< 0.05). Hepatic TG content dropped, while TC and CE increased (Fig. 4H - K, *P*< 0.05). Plasma lipid profiles improved, with elevated HDL-C and reduced LDL-C and TG (Fig. 4L - O, *P* < 0.05 or *P* < 0.01), alongside lower plasma AST and ALT levels (Fig. 4P and Q, *P*< 0.05).

### GML alters plasma metabolite profiles

**Fig. 5:**
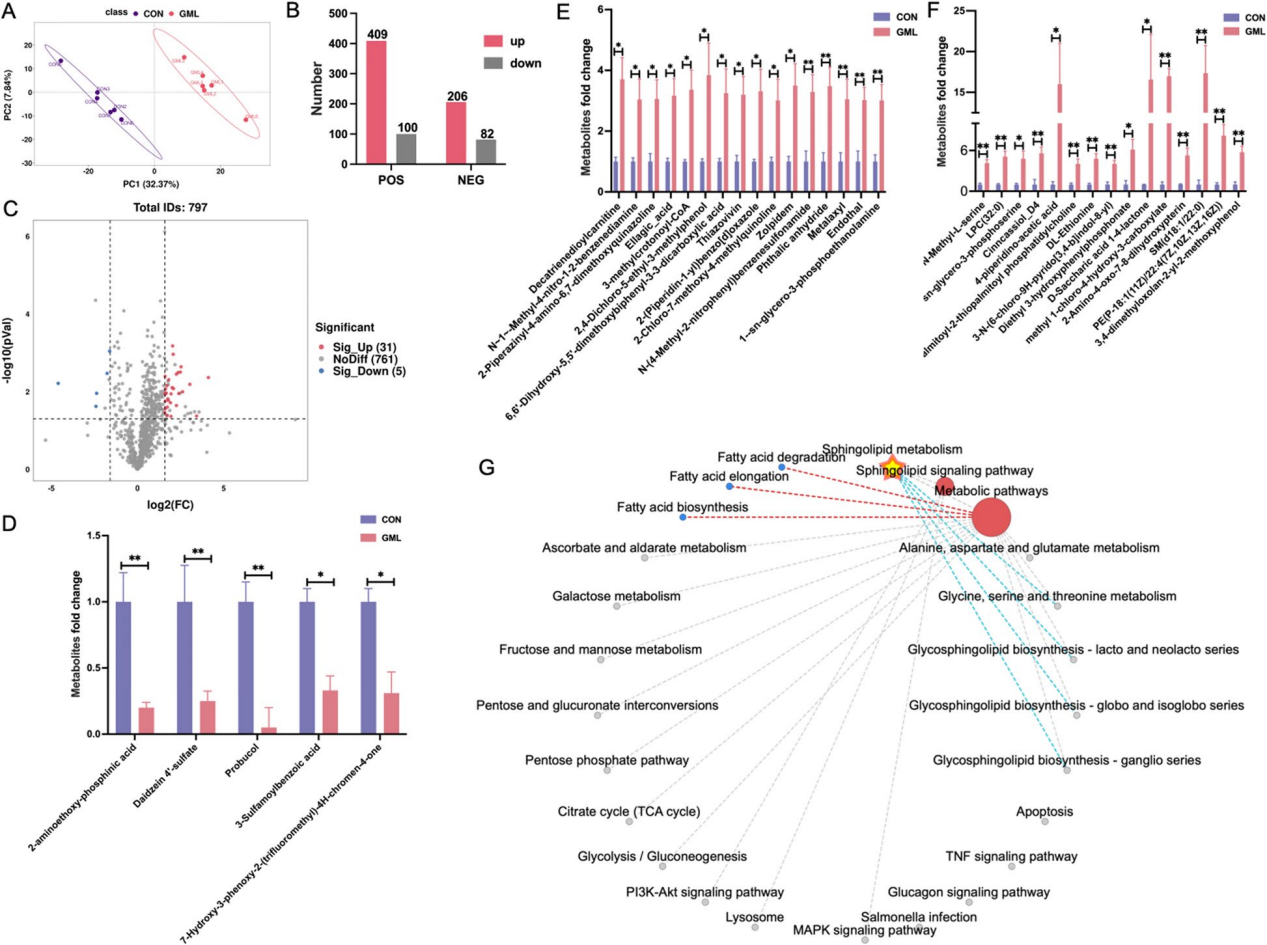
Effects of GML on plasma metabolites in heat stressed laying hens. **A** Partial least squares discriminant analysis (PLS-DA) score plot of two - group samples. **B** Number of metabolites in positive and negative ion mode. **C** Volcano map of differentially regulated metabolites. **D - F** Thirty-one significantly different metabolites screened. **G** Enriched metabolic pathways related network diagram. The *P* value is calculated by student's *t* test and two-tailed. Data are presented as mean ± SEM, *n*=6. * *P*<0.05, **P<0.01

Partial Least Squares Discriminant Analysis (PLS-DA) distinguished plasma metabolite profiles between groups (Fig. 5A). In positive ion mode, 409 metabolites were upregulated and 100 downregulated; in negative mode, 206 were upregulated and 82 downregulated (Fig. 5B). Filtering (VIP>1.5, *P* < 0.05, FC > 3 or FC < 0.33) identified 36 differential metabolites, with 31 increased (e.g., 4-piperidino-acetic acid, SM d18:1/22:0) and 5 decreased (e.g., Probucol) (Fig. 5C - F). Sphingolipid metabolism emerged as the primary enriched pathway, followed by fatty acid metabolism (Fig. 5G).

### GML modulates hepatic lipid metabolism

**Fig. 6:**
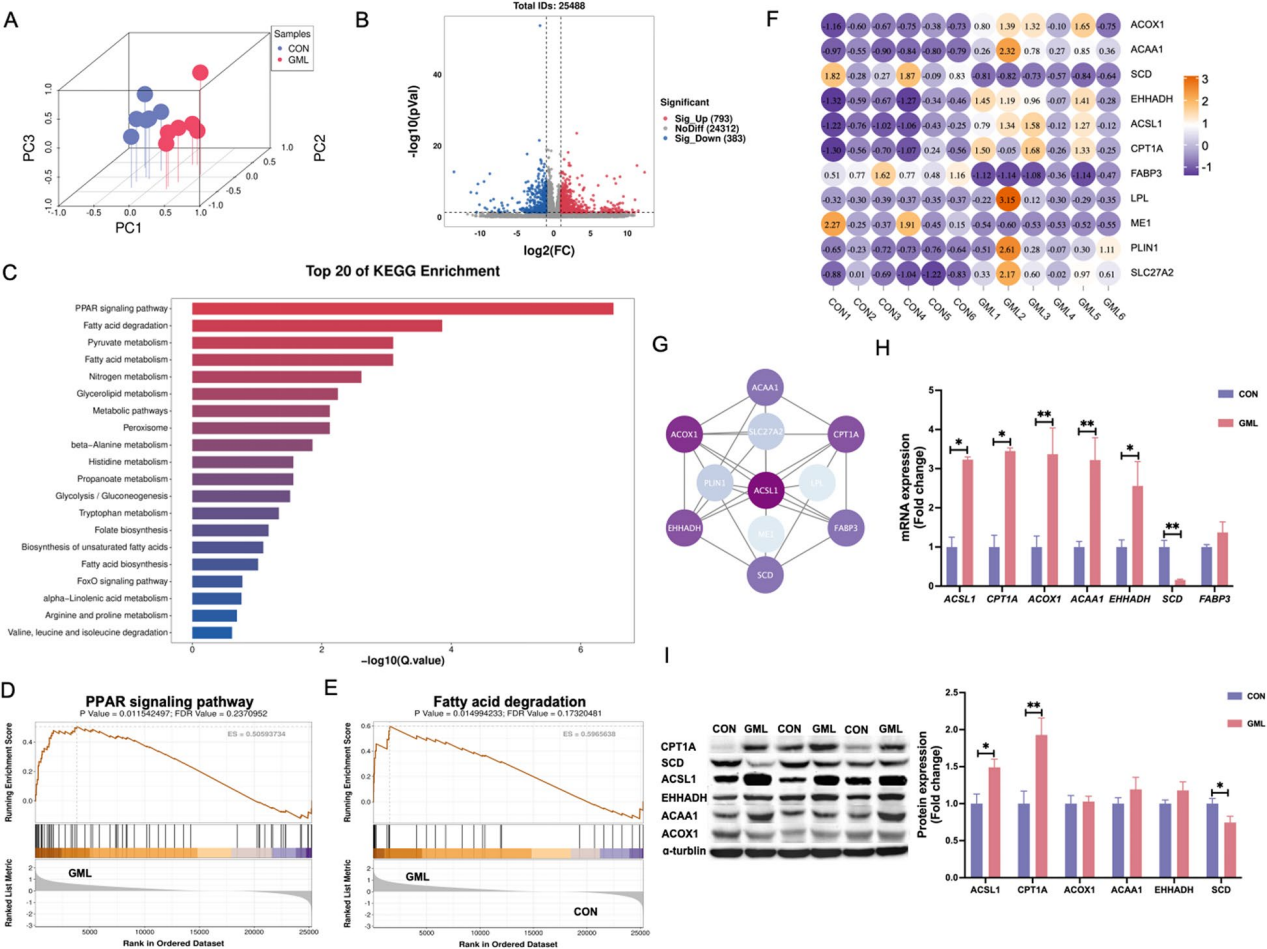
Hepatic transcriptomics. **A** 3D Principal component analysis (PCA) score plot of two - group samples. **B** Volcano plot of differentially expressed genes. **C** The top 20 significantly enriched KEGG pathways. **D** The Gene Set Enrichment Analysis (GSEA) of PPAR signaling pathway. **E** The Gene Set Enrichment Analysis (GSEA) of fatty acid degradation. **F** Heatmap of the differentially expressed genes. **G** Protein-protein interaction (PPI) network of the differentially expressed genes. **H** qPCR to verify the expression of differential genes. **I** Western blot analysis of the differentially expressed proteins. The *P* value is calculated by student's *t* test and two-tailed. Data are presented as mean ± SEM, *n*=6. * *P*<0.05, **P<0.01

Hepatic transcriptomes separated distinctly by PCA (Fig. 6A). Of 25,488 sequences, 793 genes were upregulated and 383 downregulated (|Log2 FC| > 1, *P* < 0.05; Fig. 6B). KEGG enrichment pathway of genes with significant difference found that the pathway with the top 20 enrichment were PPAR signaling and Fatty acid degradation pathway (Fig. 6C). GSEA analysis of KEGG enrichment pathway showed that the expression of PPAR signaling and fatty acid degradation pathway in GML group was significantly higher than that in control group (Fig. 6D and E). Key upregulated genes included *ACOX1*, *ACAA1*, *EHHADH*, *ACSL1*, *CPT1 A*, *LPL*, *PLIN1*, and *CPT1 A*; *SCD*, *FABP3*, and *ME1* were downregulated (Fig. 6F). These genes are enriched differential genes in PPAR signaling pathway, among which ACOX1, ACAA1, EHHADH, CPT1 A and ACSL1 also belong to fatty acid degradation pathway (Fig S4). Integrative analysis revealed ACSL1 as a hub gene connecting sphingolipid metabolism and PPAR signaling, followed by genes with strong associations, including *ACOX1*, *ACAA1*, *EHHADH*, *CPT1 A*, *SCD*, *FABP3* (Fig. 6G). qPCR and Western blot validated these shifts, except for FABP3. As shown in Fig. 6H, the mRNA levels of *ACSL1*, *CPT1 A*, *ACOX1*, *ACACA*, and *EHHADH* were significantly upregulated, while markedly downregulating *SCD*. As depicted in Fig. 6I, the protein levels of ACSL1, CPT1 A, were significantly upregulated, whereas SCD was substantially reduced in the GML group compared to the CON group.

### Integrated omics correlations

**Fig. 7:**
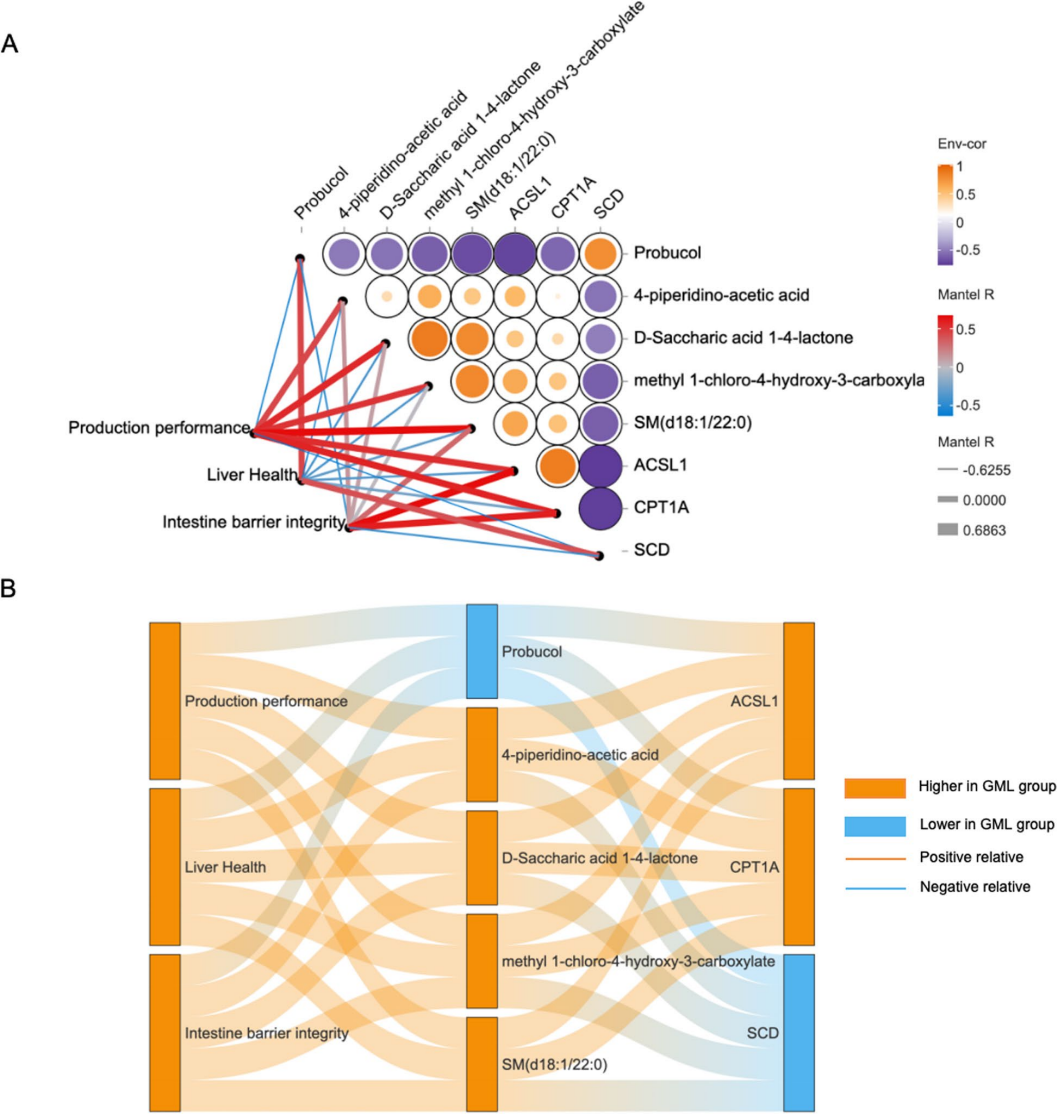
Correlation analysis. **A** Heat map of correlation between key differential genes, key differential metabolites and health indicators. **B** Sankey diagram of key differential genes, key differential metabolites and health indicators. *n*=6

Metabolomic and transcriptomic data showed strong concordance (Fig. S3A). LPC (32:0) positively correlated with *ACOX1*, *ACAA1*, *EHHADH*, *ACSL1*, and *CPT1 A*, and negatively with *SCD* (Fig. S5B and S5C). Production performance and intestinal integrity positively associated with most differential metabolites with the largest fold change and genes with significant difference, except Probucol and SCD, which correlated negatively (Fig. 7A and B). Liver health showed an inverse pattern.

## Discussion

The egg - laying industry is crucial in global agriculture for offering a sustainable source of high - quality protein. However, the growing consumer demand for eggs is being jeopardized by HS’s negative impact on laying hens, especially as global warming worsens (Khatibi et al. 2021; Mao and Lien 2017). HS disrupts homeostasis in poultry through multiple pathways, leading to impaired egg production, reduced egg quality, and metabolic imbalances (Whiting et al. 1990; Liu et al. 2020a, b). These challenges underscore the urgent need for innovative nutritional interventions to mitigate the impacts of HS on poultry health and productivity.

The hepatic regulation of lipid homeostasis is critically influenced by mitochondrial processing of macronutrients, including proteins, carbohydrates, and fatty acids. Hepatic antioxidant defense mechanisms, comprising enzymatic systems and antioxidants, effectively scavenge excessive ROS produced during this process, thereby protecting cellular macromolecules from oxidative damage and maintaining redox homeostasis (Surai et al. 2019; Emami et al. 2020). Heat stress (HS) induces oxidative stress in chickens, placing additional strain on the liver. Furthermore, HS undermines intestinal barrier integrity through various mechanisms, including pathogen - induced damage, metabolic - endocrine disturbances, and oxidative stress. Consequently, this leads to increased translocation of luminal toxins (Kikusato and Toyomizu 2023).

In this study, dietary supplementation with 325 mg/kg GML significantly increased intestinal villus height and the villus-to-crypt ratio, upregulated the expression of tight junction proteins (e.g., Occludin), and enhanced intestinal absorption capacity. Long term feeding of GML did not have toxic effects on laying hens. Previous studies reported that feeding 40 week old hy line Brown laying hens 150~300 mg/kg GML for 24 weeks improved production performance and adjusted the structure of intestinal flora (Liu et al. 2020a, b). High dose of 1600 mg/kg can significantly improve liver lipid metabolism in HFD C57BL/6 J mice (Zhao et al. 2020). Previous studies have demonstrated that GML enhances intestinal barrier function by modulating gut microbial ecology through bile acid - mediated mechanisms and suppresses inflammatory responses (van der Aar et al. 2017; Mo et al. 2019). The enhanced gut integrity likely contributed to improved nutrient absorption and production performance (Cui et al. 2023). Supplementation with GML at 1000 mg/kg demonstrated significant effects on intestinal morphology and function, including increased villus height and villus/crypt ratio, elevated ileal mucosal factor levels, and enhanced *Occludin* expression, all contributing to improved nutrient absorption (Cui et al. 2023). Maintaining intestinal barrier integrity limits hepatic translocation of gut-derived endotoxins like LPS, preventing chronic inflammatory insults that may lead to hepatic steatosis or fibrosis (Mo et al. 2021). Upregulation of the expression of intestinal barrier function related proteins ZO1 and Occludin cannot directly promote nutrient absorption, but can maintain intestinal health by increasing the activity of intestinal immunoglobulin IgA, IgG, IgM, and digestive enzymes, and maintaining the normal structure and function of intestinal villi, intestinal villi are the main site for nutrient absorption, reducing the risk of harmful substances entering the bloodstream through intercellular spaces (Sahin et al. 2018; Xu et al. 2024). Compared to low dose GML, high dose GML is more effective in improving intestinal absorption capacity, which is consistent with the results of this experiment. The addition of GML alters the metabolic efficiency of carbohydrates, improves the composition of the gut microbiota, and affects the metabolism and function of microorganisms, thereby influencing the nutrient absorption and production performance of animals (Mo et al. 2019; Zhao et al. 2019).

Medium chain monoglycerides (MCMs) and their hydrolysis products, medium chain fatty acids (MCFAs), can directly penetrate the intestinal cell membrane and enter cells through a simple diffusion method, without relying on fatty acid binding proteins (FABPs) or fatty acid transporters (such as CD36), which simplifies the absorption process (Yang et al. 2024). Moreover, short carbon chain medium chain fatty acids like GML (such as C8 - C12) have small molecular weight, low polarity, and faster transmembrane diffusion rate (Szabó et al. 2023), accelerating the body’s absorption of GML and making it easier for them to have biological effects on the body. In this study, GML supplementation effectively alleviated liver damage, as evidenced by a significant reduction in NAS (nonalcoholic fatty liver disease activity score). The improvement in liver health was accompanied by enhanced antioxidant capacity, as indicated by increased activities of SOD, T-AOC, and GSH-Px, and reduced levels of MDA in serum and liver tissue (Wang et al. 2024). The rise in SOD and GSH-Px activities alongside increased LPC 32:0 levels points to a three - tiered antioxidant approach: (i) direct ROS scavenging by GML derived metabolites, (ii) Nrf2 mediated boosting of antioxidant defenses, and (iii) sphingolipid aided membrane stabilization. This multi - layer system effectively curbs MDA generation. The upregulation of ACSL1 and other β-oxidation related genes shows GML mediated enhanced lipid breakdown, matching the lower hepatic lipid accumulation. These findings are consistent with previous reports on the ability of medium-chain fatty acids to enhance lipid transport while reducing serum triglycerides and cholesterol, reinforcing the lipid-lowering properties identified in this investigation (Shokrollahi et al. 2014; Wang et al. 2015). HS compromises intestinal barrier integrity in poultry, facilitating portal vein-mediated translocation of bacterial lipopolysaccharide, thereby exacerbating hepatic injury (Li et al. 2025). The addition of GML does not increase the liver burden, the addition of GML leads to a decrease in the content of ALT, and the function of the liver is not affected by it (Seleem et al. 2016). The portal venous system directly transports gut derived substances to the liver. In return, the liver secretes bile and immunoglobulins to adjust the intestinal environment. This creates a two - way communication between the liver and gut microbiota (Albillos et al. 2020). The enhancement of intestinal barrier function has a direct promoting effect on liver health (Chopyk and Grakoui 2020), which is consistent with the results in this study. These findings jointly confirm the beneficial effect of GML on the gut liver axis in chickens. The observed enhancement in oviposition performance and egg quality mediated by GML is primarily attributed to its modulatory effects on intestinal and hepatic functions. As a natural lipid compound, GML contributes to improved immune competence in layers, resulting in reduced disease susceptibility and enhanced production efficiency (Jackman et al. 2020). Dietary supplementation of 100–400 mg/kg GML significantly enhanced reproductive performance in Hy-Line Brown layers, manifesting in increased egg yield and improved ovum characteristics. Notably, positive changes were observed in shell integrity, protein quality, yolk pigmentation, and Haugh unit values (Liu et al. 2020a, b; Wang et al. 2024). In this experiment, the optimal dosage of GML in the feed for laying hens was determined to be 325 mg/kg. The fundamental factors leading to changes in GML dose-response are the differences in laying hen age and changes in rearing environment.

LPC is commonly used as a biomarker for diseases, and a decrease in LPC is often associated with vascular aging and impaired mitochondrial oxidative capacity (Semba et al. 2019; Polonis et al. 2020), In this study, the significant increase in plasma LPC driven by GML’s antioxidant boost and lipid transport acceleration. LPC (32:0) shuttling in the gut - liver axis forms a compensation loop for NAD+ synthesis. DL ethionine increases NAD levels in the liver (Kröger et al. 1982), and inhibit the absorption of lipids by the small intestine, thereby regulating the lipid level in the body (Kessler et al. 1969). In this study, DL-ethionine is strongly correlated with the fatty acid beta-oxidation - related gene *ACAA1*. This enzyme is crucial for peroxisomal fatty acid β-oxidation, converting 3-ketoacyl-CoA into acetyl-CoA and acyl-CoA. These products then drive fatty acid elongation and breakdown pathways (Wang et al. 2021). 4-piperidino-acetic acid has been linked to improved, selective human intestinal carboxylesterase inhibitors, demonstrating antitumor activity (Hicks et al. 2009). D-saccharic acid 1–4-lactone can inhibit liver injury caused by hyperglycemia (Bhattacharya et al. 2013a, b), moreover, it ameliorates hyperglycemia-induced oxidative stress and inflammation via modulation of Nf-κB and PKC signaling pathways, thereby exerting protective effects against metabolic dysfunction (Bhattacharya et al. 2013, Bhattacharya et al. 2013). The liver health of GML group has a strong positive correlation with D-Saccharic acid 1–4-lactone. SM (d18:1/22:0) is a ubiquitous sphingolipid, which is abundantly found in the plasma membrane and lipoproteins and is closely associated with hepatic steatosis (Li et al. 2014), SM (d18:1/22:0) was positively correlated with liver health, and the metabolic homeostasis of SM was also regulated by sphingomyelinase (Marí and Fernández-Checa 2007).

In this study, differentially expressed pathways primarily centered on peroxisome proliferator activated receptor (PPAR) - mediated signaling and fatty acid catabolism. Fatty acid homeostasis is regulated by two opposing transcriptional networks: PPARα driven fatty acid breakdown and LXR dependent SREBP-1c controlled de novo lipogenesis (Ide et al. 2003). The PPAR signaling cascade serves as a pivotal regulator of hepatic lipid homeostasis. Hepatic steatosis manifests when lipid accumulation surpasses catabolic capacity, characterized by an imbalance between fatty acid uptake and de novo lipogenesis versus fatty acid β-oxidation and lipid export. NAFLD pathogenesis involves upregulated hepatic fatty acid uptake and enhanced de novo lipogenesis, with compensatory stimulation of fatty acid β-oxidation proving insufficient to maintain lipid homeostasis (Ipsen et al. 2018). GML treatment markedly elevated cecal and fecal concentrations of acetate, propionate, and butyrate, These SCFAs, functioning as signaling molecules, subsequently activated hepatic AMPK and PPARα pathways, enhancing lipid oxidative metabolism (Mo et al. 2019). Transcriptional profiling revealed predominant upregulation of genes associated with fatty acid β-oxidation pathways. Concurrently, SCD downregulation correlated with reduced lipid accumulation. SCD, a key regulator of saturated and unsaturated fatty acid synthesis, shows inverse regulation by polyunsaturated fatty acid levels, enhanced SCD activity is linked to suppressed lipid oxidation and promoted fatty acid synthesis (Bjermo and Risérus 2010; Flowers and Ntambi 2009). *ACSL1* is the core target gene in this experiment. *ACSL1* can guide fatty acids into mitochondria for oxidation and promote fatty acid beta oxidation (Young et al. 2018; Ellis et al. 2010). The up regulation of fatty acid β-oxidation promotes the process of fatty acid to energy conversion. In this study, liver health and the down-regulation of liver triglycerides are closely related to *ACSL1*. GML enhances intestinal barrier function, antioxidant capacity, and lipid metabolism in laying hens under heat stress, leading to improved egg production and quality, and highlights its potential as a natural intervention to mitigate heat stress impacts in poultry production.

## Conclusion

In summary, this study provides comprehensive evidence for the efficacy of GML in mitigating HS in laying hens. By enhancing intestinal barrier integrity, modulating hepatic lipid metabolism via the PPAR signaling pathway, and improving antioxidant capacity, GML supplementation at an optimal dosage of 325 mg/kg significantly improved egg production and quality. These findings highlight the potential of GML as a multifunctional feed additive for sustainable poultry production under HS conditions. Future studies should investigate the long-term effects of GML supplementation, its interactions with other feed additives, and its applicability across different poultry species.

## Materials and methods

### Breeding and management of animals

A total of 504 healthy 43-week-old Hy-line Brown laying hens exhibiting comparable egg production rates during peak laying period, were randomly distributed across four experimental groups, with six replicate pens (21 hens per pen). HS conditions were maintained at 32±1℃ (RH 65±5%) for 8 h/day, with thermal humidity index (THI)≥78, exceeding the comfort threshold (THI = 72) for laying hens. The control group received basal diet while experimental groups were supplemented with GML at graded levels of 65, 195, or 325 mg/kg feed, which was received from Ecolex Sdn. Bhd., Malaysia. The 10-week trial consisted of 2-week adaptation and 8-week experimental phases, with daily feed restriction to 130 g/bird and ad libitum water access. Management conditions: 16-hour photoperiod, free drinking water, quantitative diet. Egg production rate (%) and egg weight (g) were recorded daily, with egg quality parameters analyzed at 4-week intervals throughout the experimental period. The composition and nutritional level of the basic diet are shown in Table S1.

### Egg quality

Egg quality parameters were assessed at 4-week intervals using 50 eggs per group. Measurements included shell weight (g), shell ratio (%), yolk weight (g), yolk ratio (%), albumen weight (g), albumen ratio (%), albumen height (mm), yolk color, haugh unit, shell strength (kg/cm^2^), and shell thickness (mm). Shell strength was determined using an impact resistance tester (WW-2 A, Nanjing Soil Instrument Factory Co., Ltd) to measure maximum fracture resistance. Shell thickness measurements (mm) were obtained from equatorial, blunt, and sharp regions using a micrometer screw gauge (values reported as mean of three positions, excluding shell membranes). All other parameters were analyzed through nondestructive measurement using a multifunctional egg analyzer (EMT-5200, Robotmation Co. Ltd., Tokyo, Japan). Numerical results represent triplicate determinations per egg under standardized conditions (25℃, 60 ± 5% RH).

### Sample collection

Sample collection was performed in control and 325 mg/kg GML groups based on production performance and egg quality analyses. Ten hens per group were randomly selected from six replicate pens for biological sampling. Blood collected from wing veins via venipuncture was deposited in non-anticoagulant tubes, centrifuged (3,000 rpm, 10 min, 4℃), and plasma aliquots were immediately maintained on ice. Liver and small intestinal tissues were rinsed with physiological saline, divided into two batches: one immersed in 4% paraformaldehyde for histological fixation at 4℃ and the other flash-frozen in liquid nitrogen for cryopreservation.

### Plasma biochemistry and antioxidant

Serum biochemical indexes, including total cholesterol (TC), total triglycerides (TG), high-density lipoprotein (HDL-C), low-density lipoprotein (LDL-C), alanine aminotransferase (ALT), aspartate aminotransferase (AST), were determined by a fully automatic biochemical analyzer (Hitachi 7020, Tokyo Japan). The kits were from Sanhe instrument company (Nanjing, CN).

### Plasma metabolomics

Plasma samples were prepared under strict pre-analytical standardization protocols. Whole blood collected in EDTA-containing vacutainers (Becton Dickinson, USA) underwent centrifugation (3,500 rpm, 15 min, 4℃) within 60 min post-collection. Separated plasma aliquots were stored in cryovials (Eppendorf, Germany) at −80℃ until analysis. Metabolite extraction involved mixing 100 μL plasma with 400 μL ice-cold methanol/water (4:1 v/v; LC-MS grade, Fisher Scientific) containing 0.1% formic acid, followed by vortexing (2 min) and centrifugation (16,000 rpm, 15 min, 10℃). The supernatant was filtered (0.22 μm PTFE membranes, Millipore) prior to LC-MS analysis, using a ZORBAX Eclipse Plus C18 column (2.1 × 100 mm, 1.8 μm; Agilent Technologies) thermostated at 40℃. Mobile phase A consisted of 0.1% formic acid in water and phase B contained 0.1% formic acid in acetonitrile (both Optima™ LC/MS grade). The 26-min gradient program progressed from 5% B to 100% B over 18 min, held for 3 min, then re-equilibrated for 5 min at 5% B, with a flow rate of 0.3 mL/min. High-resolution mass spectrometry was performed on a Q Exactive HF-X Hybrid Quadrupole-Orbitrap system (Thermo Fisher Scientific) operating in positive and negative ionization modes. Quality control samples (pooled plasma aliquots) were injected every 10 samples to monitor instrument performance (RSD < 15% for 92% of features in QC samples). Batch-to-batch variation was minimized through randomized sample analysis order and column conditioning protocols.

### Intestinal barrier function

Intestinal barrier function assessments included serum diamine oxidase (DAO, MBE11198) and D-lactate (D-LA, MBE11961) concentrations were measured using ELISA kits (MALLBIO, Beijing, CN). Tight junction gene expression (*ZO1* and *Occludin*) in ileum and jejunum tissues was analyzed by quantitative PCR (SYBR Green-based detection, CFX96 system, Bio-Rad). Protein localization of *ZO1* and *Occludin* in paraformaldehyde-fixed intestinal sections was visualized through immunohistochemistry. Tissue samples underwent standardized fixation in 4% neutral buffered formalin (NBF; pH 7.4) for 24 h at room temperature, followed by sequential dehydration in graded ethanol solutions (70%, 80%, 95%, and 100%) and paraffin embedding. Tissue sections (4 μm) were placed onto poly-L-lysine-coated glass slides. After standard deparaffinization in xylene and rehydration through graded alcohols, heat-induced epitope retrieval was carried out by incubating slides in preheated 10 mM sodium citrate buffer (pH 6.0) at 95℃ for 20 min using a pressurized antigen retrieval system. Endogenous peroxidase activity was quenched with 3% hydrogen peroxide in methanol for 15 min at room temperature. Non-specific binding was blocked with 5% normal goat serum (Vector Laboratories) in PBS containing 0.1% Tween-20 (PBST) for 1 h at 37℃. Primary antibodies diluted 1:200 was applied and incubated overnight at 4℃ in a humidified chamber. After three washes with PBST, sections were incubated with corresponding horseradish peroxidase (HRP)-conjugated secondary antibodies (1:500 dilution) for 1 h at 37℃. Signal detection was achieved using 3,3’-diaminobenzidine (DAB; Dako) substrate under microscopic monitoring, followed by counterstaining with Mayer’s hematoxylin. Antibody information was in Table S3.

### Liver health

Hepatic analyses included morphometric indices, biochemical profiling, and histopathological evaluations. The hepatosomatic index (liver-to-body weight ratio) was calculated from aseptic organ dissection measurements. Lipid metabolism parameters triglyceride (TG, A110-1-1, Nanjing Jiancheng Bioengineering Institute, CN), total cholesterol (TC, E1016-105, Beijing Applygen Technologies Inc., CN), free cholesterol (FC, E1015-105, Beijing Applygen Technologies Inc., CN), and esterified cholesterol (EC = TC - FC) were determined in liver homogenates. Antioxidant status was assessed through hepatic levels of T-AOC, T-SOD, MDA, CAT, GSH-Px and GSH, all kits from Nanjing Jiancheng Bioengineering Institute (China). Paraformaldehyde-fixed liver sections (4%, 24 h) underwent hematoxylin-eosin and Oil Red O staining for steatosis visualization, with NAFLD Activity Score (NAS) quantification - a validated histopathological grading system assessing steatosis (0–3), lobular inflammation (0–3), and hepatocellular ballooning (0–2).

### Liver transcriptomics

Liver specimens were processed for transcriptomic profiling by Biotree Biomedical Technology Co., Ltd (Shanghai, China). Total RNA isolation was performed using TRIzol reagent (Thermo Fisher, 15596018), following the manufacturer’s instructions. RNA quality control included concentration measurement using Nanodrop ND-1000 spectrophotometer (Nanodrop, Wilmington, DE, USA) and integrity assessment with Bioanalyzer 2100 (Agilent, CA, USA). Only samples meeting the following criteria were processed further: RNA concentration >50 ng/μL, RIN >7.0, and total RNA yield > 1 μg. For library preparation, PolyA+ mRNA was enriched using oligo(dT) magnetic beads (Dynabeads Oligo(dT), 25–61005, Thermo Fisher) through two rounds of purification. The mRNA was then fragmented at 94℃ for 5–7 min using NEBNext Magnesium RNA Fragmentation Module (E6150S). First-strand cDNA synthesis was conducted with SuperScript II Reverse Transcriptase (1896649, Thermo Fisher), followed by second-strand synthesis using E. coli DNA polymerase I (NEB, M0209) and RNase H (NEB, M0297) in the presence of dUTP Solution (Thermo Fisher, R0133). After end repair and A-tailing, adaptors were ligated, and size selection was performed using magnetic beads. Subsequent UDG (NEB, M0280) treatment enabled strand-specific library preparation. PCR amplification was carried out as follows: 95℃ for 3 min; 8 cycles of 98℃ for 15 s, 60℃ for 15 s, 72℃ for 30 s; final extension at 72℃ for 5 min. The resulting libraries, with average fragment sizes of 300 ± 50 bp, were sequenced on Illumina Novaseq 6000 platform (PE150) following manufacturer’s protocols. Hepatic DEGs were defined as genes with |log2(fold change) | > 1, and *P* < 0.05 using DESeq2. Protein protein interaction (PPI) networks provide a comprehensive framework for understanding the complex molecular interactions in biological systems. These networks are graphical representations, where nodes represent individual genes/proteins, and edges represent the interactions between them that have been verified or predicted by experiments. PPI network topology provides important insights into differential gene function, pathway organization and cellular processes.

### Quantitative polymerase chain reaction

For RNA extraction, tissue homogenates were processed with TRIzol reagent (Thermo Fisher Scientific, #15596018) after DNase I digestion (Qiagen, #79254) to remove potential genomic DNA. RNA purity was verified by NanoDrop 2000 measurements (A260/A280 > 1.9) and electrophoretic analysis on 1% agarose gels. cDNA synthesis was carried out with PrimeScript RT Master Mix (Takara, #RR036 A) using 1 μg total RNA as template, with the following thermal profile: 15 min at 37℃ for reverse transcription, then 5 s at 85℃ for enzyme inactivation.

Gene-specific primers (Supplementary Table S2) were designed using NCBI Primer-BLAST with stringent parameters: amplicon size 90 - 150 bp, melting temperature (Tm) 60℃ ± 2℃, and avoidance of secondary structures (OligoAnalyzer Tool, IDT). Primer specificity was confirmed by melt curve analysis (single peak within 0.5℃ variance) and agarose gel verification of single-band amplicons. Quantitative PCR was performed using PowerUp SYBR Green Master Mix (Applied Biosystems, #A25742) in 10 μL reactions containing 5 μL master mix, 0.5 μM of each primer, and 20 ng cDNA template. Reactions were carried out on QuantStudio 6 Pro System (Applied Biosystems) with the following conditions: 2 min at 50℃ (UDG activation), 2 min at 95℃ (initial denaturation), then 40 cycles of 15 s at 95℃ and 1 min at 60℃. To ensure data reliability, each run included no-template controls (NTCs) and interplate calibrators. Relative quantification utilized the ΔΔCt method normalized to β-actin and GAPDH reference genes (geometric mean). Primer efficiencies (90–110%) were validated via dilution series (*R*^2^ > 0.99). Statistical significance (*P* < 0.05) was determined by GraphPad Prism 9.0 using one-way ANOVA with Tukey’s post hoc test, with triplicate biological replicates (*n*=3) and triplicate technical repeats. Intra- and inter-assay coefficients of variation were maintained below 5% and 12%, respectively.

### Western blot

Tissue proteins were extracted using RIPA lysis buffer (P0013B, Beyotime, CN) supplemented with protease and phosphatase inhibitor cocktails (HY-K0010, MCE, NJ, USA). Protein concentrations were determined via BCA assay (PC0020, Solarbio, CN) with bovine serum albumin standards. Samples (50 μg protein per lane) were denatured in Loding buffer at 95℃ for 5 min and resolved on 10% SDS-PAGE gels (GF1800-10, Genefist, CN) at 120 V for 90 min. After three TBST washes (10 min each), membranes were probed with HRP-conjugated secondary antibodies (goat anti-rabbit IgG, 1:5000, Abcam, #ab6721) for 2 h at ambient temperature. Chemiluminescent signals were developed using ECL Basic Plus Kit (RM00020P, Abclonal, CN) and captured on an Amersham Imager 600 (GE Healthcare). Densitometric analysis was performed with ImageJ 1.53k software (NIH), normalized to β-actin loading controls, and expressed as fold change relative to untreated controls. Antibody information was in Table S3.

### Statistical analysis

Data are presented as mean ± standard error of the mean (SEM). All analyses were performed using SPSS 20.0 software. Productiot ten performance and egg quality data differences were evaluated using one-way ANOVA, followed by Duncan’s multiple comparisons test. Other data between-group comparisons were conducted using independent two-tailed Student’s *t*-test after verification of normality (Shapiro-Wilk test, *P* > 0.05) and homogeneity of variance (Levene’s test, *P* > 0.05). The difference at *P* < 0.05 was considered statistically significant.

## Supplementary Information

Supplementary Material 1.

## Data Availability

Please contact the corresponding author who will make it available upon reasonable request.

## Abbreviations

ACSL1: Acyl-CoA Synthetase Long-Chain Family Member 1
ACOX1: Acyl-CoA Oxidase 1
ALT: Alanine Aminotransferase
AST: Aspartate Aminotransferase
CAT: Catalase
CPT1 A: Carnitine Palmitoyltransferase 1A
DAO: Diamine Oxidase
D-LA: D-Lactate
EC: Esterified Cholesterol
FABP3: Fatty Acid Binding Protein 3
FC: Free Cholesterol
GML: Glycerol Monolaurate
GSH: Glutathione
GSH-Px: Glutathione Peroxidase
HDL-C: High-Density Lipoprotein Cholesterol
HS: Heat Stress
IHC: Immunohistochemistry
LPC: Lysophosphatidylcholine
LDL-C: Low-Density Lipoprotein Cholesterol
MDA: Malondialdehyde
NAS: NAFLD Activity Score
NAFLD: Non-Alcoholic Fatty Liver Disease
PPAR: Peroxisome Proliferator-Activated Receptor
PPI: Protein-protein interaction
SCD: Stearoyl-CoA Desaturase
TC: Total Cholesterol
TG: Triglycerides
T-AOC: Total Antioxidant Capacity
T-SOD: Total Superoxide Dismutase
THI: Temperature-Humidity Index
ZO-1: Zonula Occludens-1
