# Supplementary material for: Dietary glycerol monolaurate mitigates heat stress-induced disruption of intestinal homeostasis and hepatic lipid metabolism in laying hens
Source: Stress Biol. 2025 Aug 12;5(1):49. doi: 10.1007/s44154-025-00243-8 (PMC12343427; doi:10.1007/s44154-025-00243-8)
Supplement: Supplementary file 1 — Supplementary Material 1. [file 44154_2025_243_MOESM1_ESM.docx]

**Table S1** Composition and nutrient level of basal diet for laying hens

| Ingredient | Percentage（%） | Analyzed nutrient | Content |
| --- | --- | --- | --- |
| Corn | 62.00 | Metabolizable energy (Mkal/kg) | 2.61 |
| Soybean meal | 24.00 | Crude protein (%) | 16.02 |
| Limestone | 9.00 | Lysine (%) | 0.74 |
| Premix | 4.50 | Methionine (%) | 0.38 |
| Soybean oil | 0.50 | Available phosphorus (%) | 0.31 |
|  |  | Total phosphorus (%) | 0.51 |
|  |  | Threonine (%) | 0.64 |
|  |  | Arginine (%) | 0.99 |
|  |  | Calcium (%) | 3.87 |

Note: The premix consists of 1% stone powder, 1.2% calcium hydrogen phosphate, 0.12% DL-methionine, 1% composite premix, 0.3% salt (feed grade), and 0.88% carrier.

**Table S2** Nucleotide sequences of specific primers for qPCR

| Genes | Forward primer (5'-3') | Reverse primer (5'-3') |
| --- | --- | --- |
| *ZO1* | GCAGTCCCTTACCTTTCCCC | AGGTTGATGAGGTTGCTGGG |
| *Occludin* | AGGTCTGCAACAGCATCACA | ATGCCTTCCCAAAAAGCCCT |
| *ACSL1* | ACCGGTCTGTGCGTTGTTG | CGAGCATCCTCTTCACCCTC |
| *CPT1A* | TGAGCACTCTTGGGCAGATG | TCTCCTTTGCAGTGTCCGTC |
| *ACOX1* | GGAGATCGAGGCCTTAGTGA | CTGGGTGAGAAGGGTAGGGA |
| *ACAA1* | AACAGCAAAGCTCGCGATTG | GCTTTGCCAAACCTTCCAGG |
| *EHHADH* | AGATCCTGGCCTACGTCTGT | GCTCCAACTGGCTGTGTTTG |
| *SCD* | ACCTTAGGGCTCAATGCCAC | TCCCGTGGGTTGATGTTCTG |
| *FABP3* | CCGCACCTATGAGAAGGCAT | CCCCATGAGACCACAGCATC |
| *β-actin* | TGCTGTGTTCCCATCTATCG | TTGGTGACAATACCGTGTTCA |

**Table S3** Antibody Information List

| Antibody | Dilution ratio | Serial Number | Manufacturer |
| --- | --- | --- | --- |
| ZO1 | 1 : 200 | A11417 | ABclonal |
| Occludin | 1 : 200 | A2601 | ABclonal |
| CPT1A | 1 : 1000 | A5307 | ABclonal |
| SCD | 1 : 1000 | A16429 | ABclonal |
| ACSL1 | 1 : 1000 | A16253 | ABclonal |
| EHHADH | 1 : 1000 | A5717 | ABclonal |
| ACAA1 | 1 : 1000 | A25034 | ABclonal |
| ACOX1 | 1 : 1000 | A8091 | ABclonal |
| a-turblin | 1 : 1000 | A6830 | ABclonal |


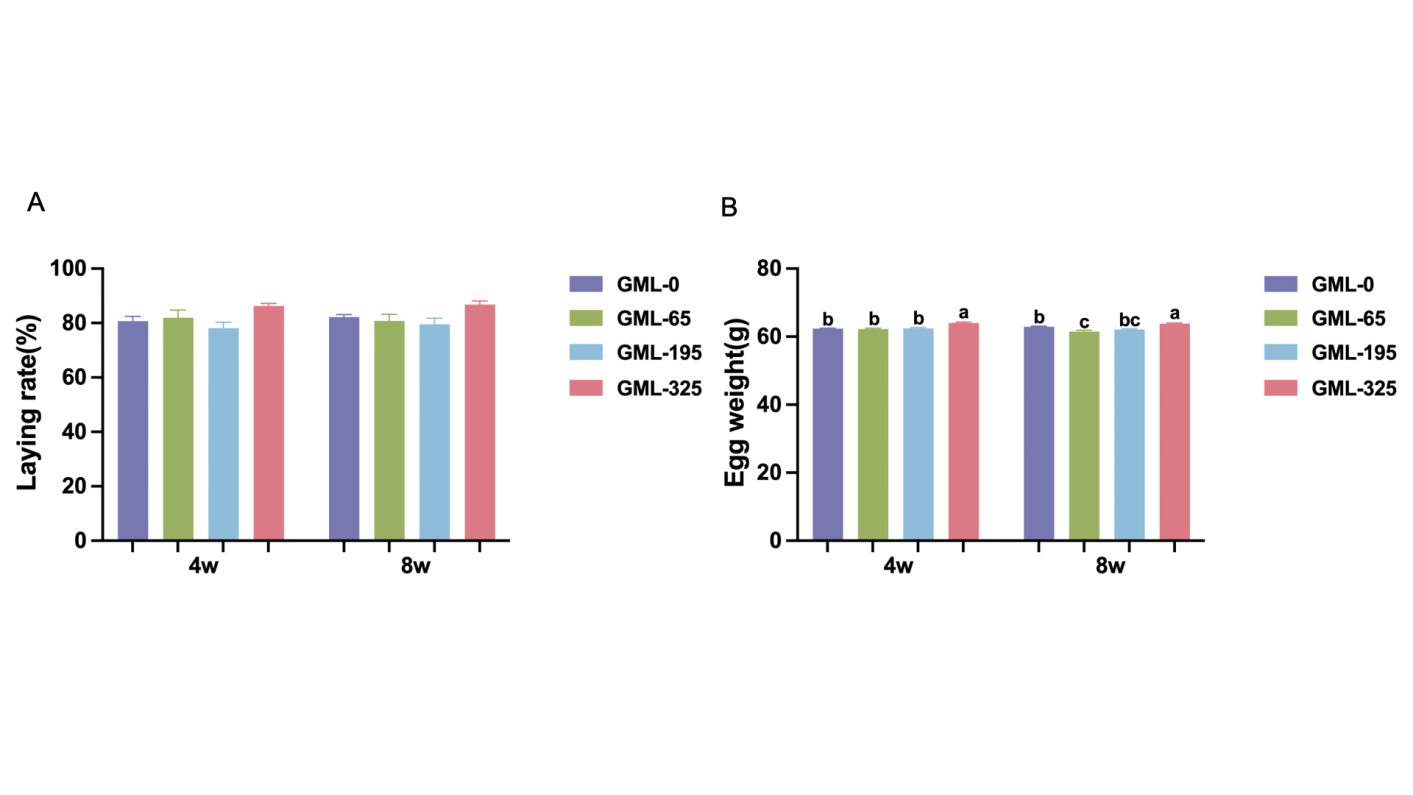


**Fig. S1** Effects of different concentrations of GML on production performance of laying hens under heat stress. **A** laying rate. **B** egg weight. Data are presented as mean ± SE, n=6.The *P* value is calculated by one-way ANOVA and duncan's multiple comparisons test. Different lowercase letters in the shoulder label indicate a significant difference (*P*＜0.05).

**
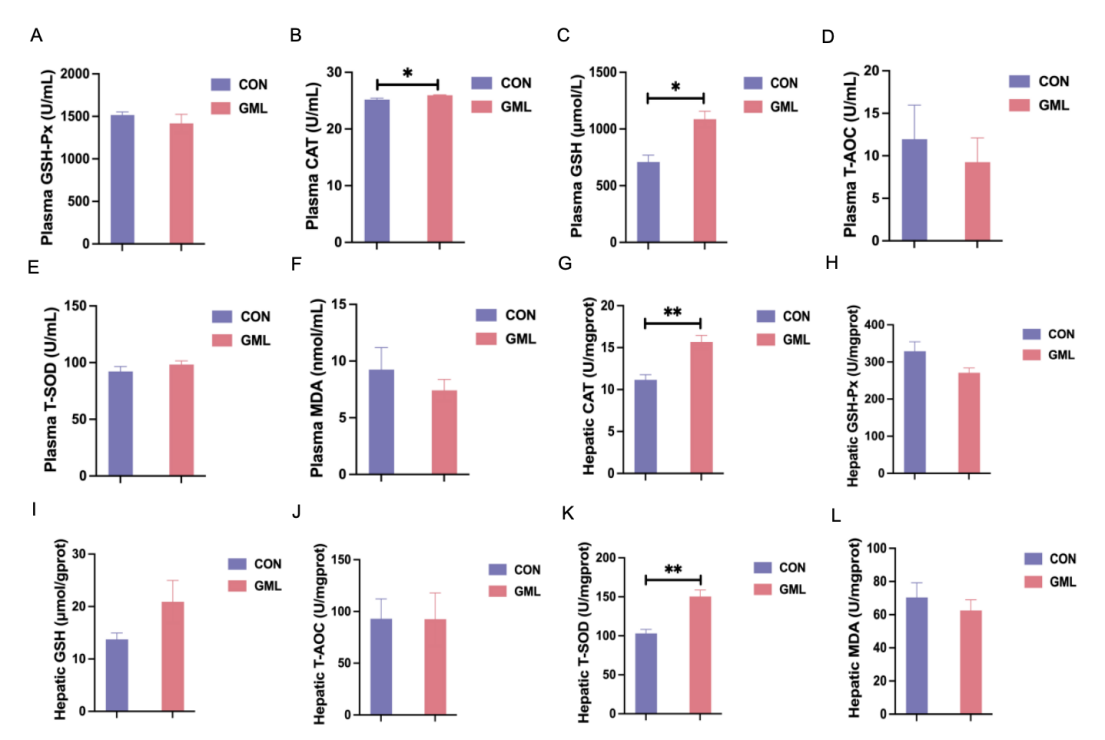
**

**Fig.S2** Effect of GML on antioxidant capacity of heat stressed laying hens. **A** Plasma GSH-Px activity. **B** Plasma CAT activity. **C** Plasma GSH concentration. **D** Plasma T-AOC. **E** Plasma T-SOD activity. **F** Plasma MDA concentration. **G** Hepatic CAT activity. **H** Hepatic GSH-Px activity. **I** Hepatic GSH concentration. **J** Hepatic T-AOC. **K** Hepatic T-SOD activity. **L** Hepatic MDA concentration. The *P* value is calculated by student t test and two-tailed. Data are presented as mean ± SEM, n=6. * *P*＜0.05, ***P*＜0.01.


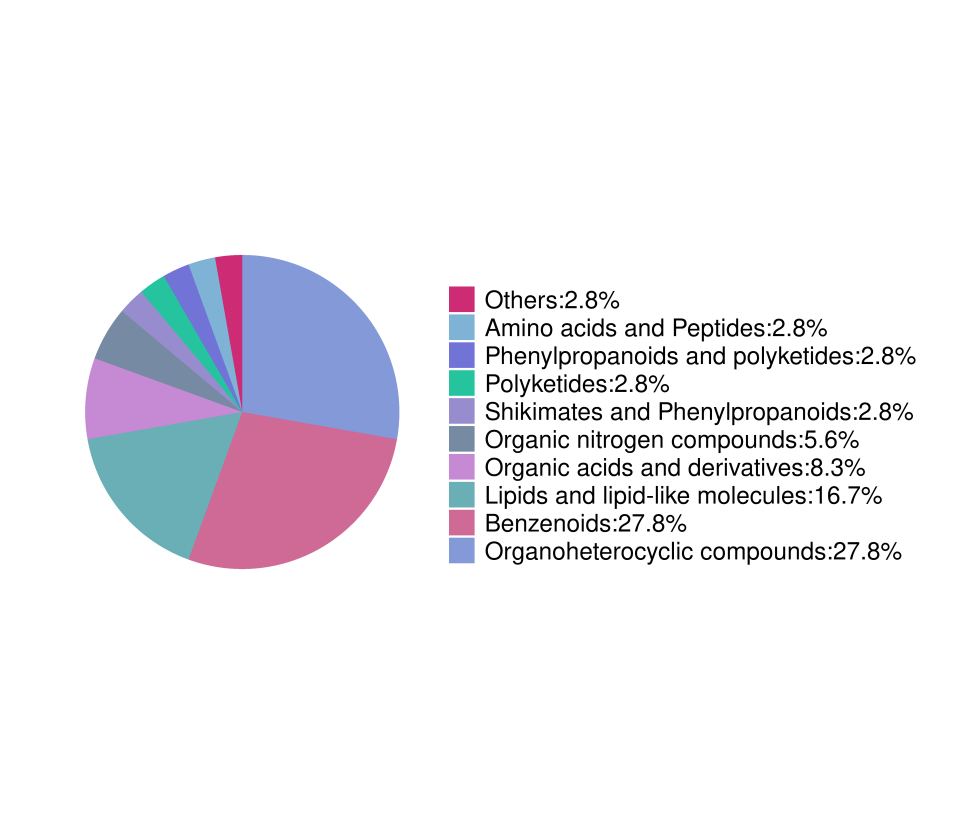


**Fig. S3** Differential metabolites pie chart.

Classification of significantly different metabolites.


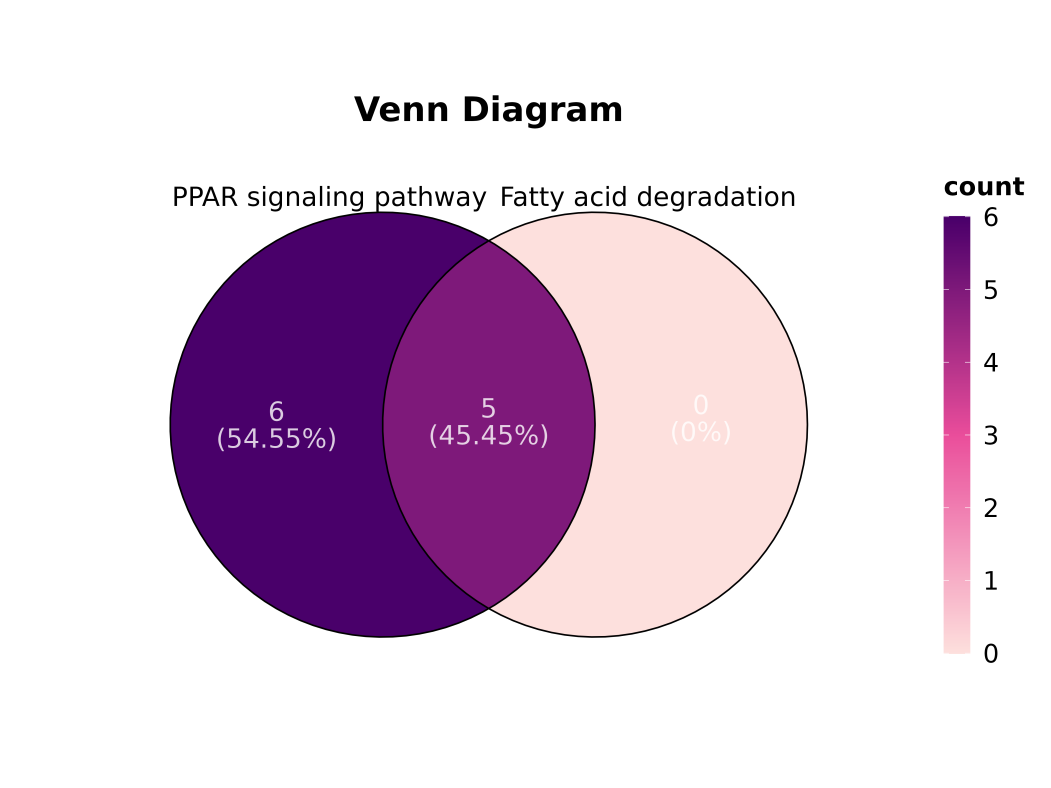


**Fig. S4** Venn diagram of differential pathways and differential genes.

PPAR signaling pathway and fatty acid degradation enrichment differential genes.

**
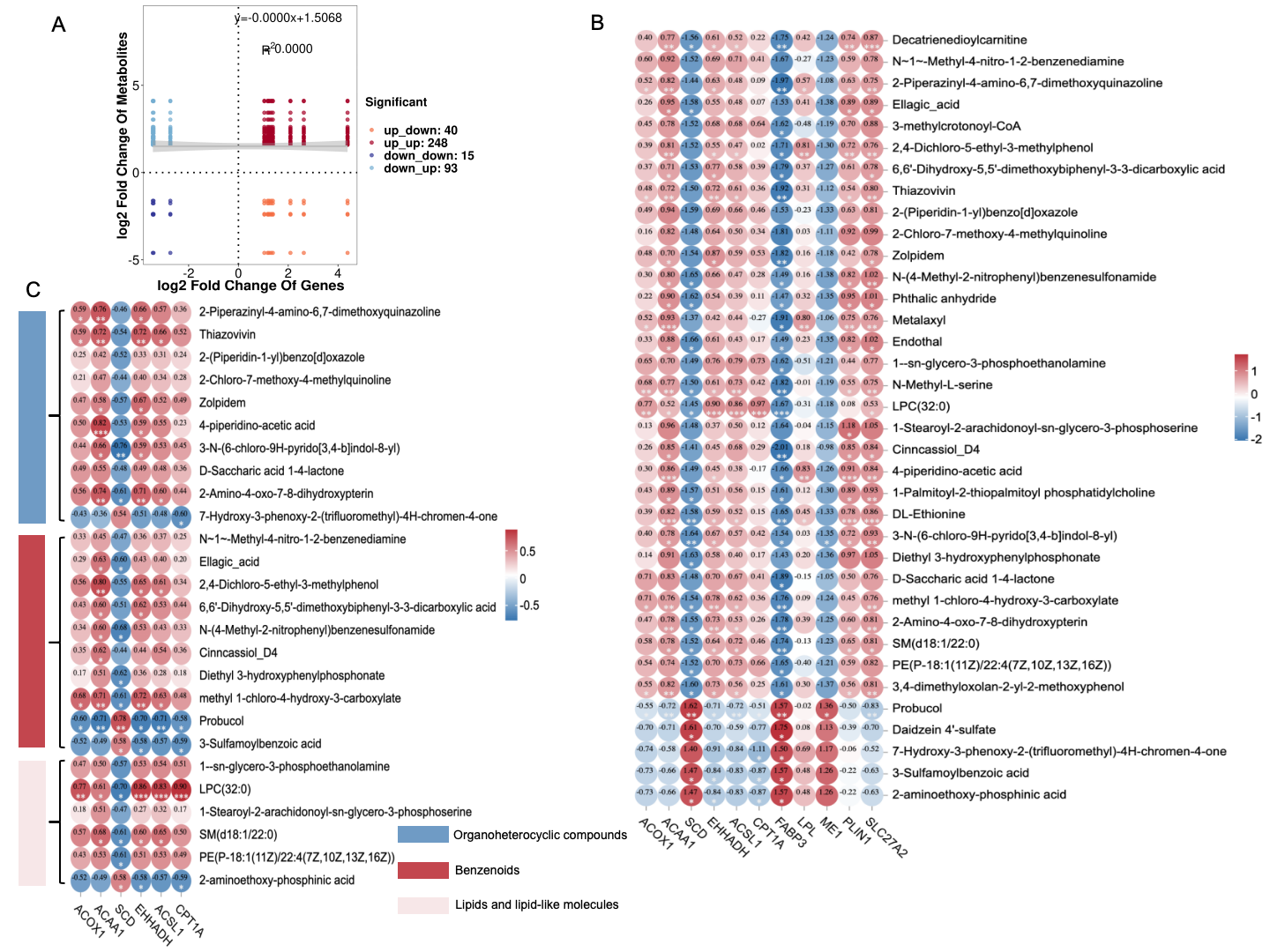
**

**Fig. S5** Heatmap of correlation between metabonomics and transcriptomics. **A** Omics correlation quadrant diagram. **B** Heatmap of differential genes and differential metabolites. **C** Heatmap of key differential genes and main differential metabolites differential genes and differential metabolites. The *P* value is calculated by student t test and two-tailed, n=6. * *P*＜0.05, ***P*＜0.01, ****P*＜0.001.
